# The Ministry of Health

**Published:** 1918-07-27

**Authors:** 


### The Hospital

The Workers' Newspaper of Administrative Medicine and Institutional Life, Administration, National Insurance and Health.

No. 1677, Vol. LXIV. SATURDAY, JULY 27, 1918. PRICE TWOPENCE

### THE MINISTRY OF HEALTH.

The discussion on the Ministry of Health in the medical press and at the Royal Society of Medicine reveals much difference of opinion amongst medical men. With but few exceptions, all agree that a new department is desirable to deal with medical matters only, and to take over from existing Government departments all the medical work at present done by them for the civil population of the kingdom. Some wish to see the matter end there, but the majority would give the new department fresh powers and expect from it a great extension of public medical work. It is over these powers and the method of their exercise that the profession is at variance. A small minority are in favour of a State Medical Service, the remainder would like to see private practice State aided to a greater or less extent, with as little interference as possible with the freedom of the medical man and of his patients. There are many who seem to expect the State to subsidise medical men without exercising any control in return for its subsidies. That is impossible, The profession cannot expect to enjoy the advantages of State aid and to continue in complete freedom. Nor can it expect a free hand, nor even a controlling influence in directing the national medical policy. Englishmen have a strong and quite sound objection to government by experts. They prefer to be ruled by men of common sense, who will listen to the expert, but will be guided in their decisions by a broader view than the expert commands.

Now is the time for the general practitioner to choose definitely which of two paths he wishes to follow. Does he wish medical practice to remain as it is now, in the main, a matter of individual effort and competition, or does he wish it to become a National Medical Service? If his inclinations lead him to answer yes to the first question, then he must try to influence our law-makers so that the Ministry of Health may be a department merely to coordinate the public medical work at present done by many Government departments. He must watch jealously any extension of its powers, and confine them as far as possible to whole-time services. All other schemes lead more or less quickly to a National Service. The proposals of the British Medical Association, for instance, are a long step towards it. Practically the whole profession would come under the control of the Ministry of Health and the Local Administrative Health Committees. The subsidised voluntary hospitals would approximate more and more closely to the State-maintained hospitals with every increase of their subsidies. The greater the medical work undertaken by the State, the less will be the scope for voluntary efforts.

The opposition to a State Service comes from the private practitioner. Those already in public Services, the whole-time medical officers of health, Poor-Law medical officers, tuberculosis medical officers, etc., are almost unanimously in favour of it, when they take any interest at all in the matter. And it is interesting to note that a letter was read at one of the meetings at the Royal Society of Medicine from a man holding a temporary commission in the R.A.M.C., in which he said that practically all his colleagues were hoping for the formation of a State Medical Service. Most of these are men who have had experience of work under both systems, and if the writer of the letter really represents the view of the majority, the testimony is of the greatest value. Opponents of a State Medical Service say that it would destroy the freedom of action of the medical man, would eliminate all competition, remove all incentive for a man to do his best work, crush out his desire for research and self-improvement, and make him a mere official striving to do only the minimum of work that would enable him to escape the censure of his superior officers. Are these statements true? Is the private practitioner more free and untrammelled than the medical man in a service with his definite duties and leisure? Are competition and the desire of monetary gain the only incentives to research and advancement? Is there no pleasure in the work itself, no joy in discovery, no stimulus in the desire to place before fellow-workers the results of one's own work? Is medical work so unpleasant that men would wish to scamp it unless every item brought a payment in cash? Of course if a man does not love his work for its own sake, then he is not likely to do his best as a salaried official, but, on the other hand, he is not likely to be much of a success under any system. It must be remembered that gross neglect of duty would render the defaulter liable to expulsion from the Service. Again, competition would not be entirely eliminated under any system of State Service. There would always be a certain amount of private practice which would go to those who competed for it, and a capitation system of payment would give the most popular and successful men in any area the biggest income.

The discussions of the last few months have given medical men the means to form their judgment. It is now for them to give their decision. If they are indifferent, if they take no steps to influence forthcoming legislation, they will have no right to grumble if they find introduced drastic changes to which they object.

